# Rehabilitative exercise and spatially patterned nanofibrillar scaffolds enhance vascularization and innervation following volumetric muscle loss

**DOI:** 10.1038/s41536-018-0054-3

**Published:** 2018-09-17

**Authors:** Karina H. Nakayama, Cynthia Alcazar, Guang Yang, Marco Quarta, Patrick Paine, Linda Doan, Adam Davies, Thomas A. Rando, Ngan F. Huang

**Affiliations:** 1 Veterans Affairs Palo Alto Health Care System, 3801 Miranda Avenue, Palo Alto, CA 94304 USA; 2 The Stanford Cardiovascular Institute, Stanford University, Stanford, CA 94305 USA; 3 Department of Cardiothoracic Surgery, Stanford University, Stanford, CA 94305 USA; 4 Department of Neurology and Neurological Sciences, Stanford University, Stanford, CA 94304 USA

## Abstract

Muscle regeneration can be permanently impaired by traumatic injuries, despite the high regenerative capacity of skeletal muscle. Implantation of engineered biomimetic scaffolds to the site of muscle ablation may serve as an attractive off-the-shelf therapeutic approach. The objective of the study was to histologically assess the therapeutic benefit of a three-dimensional spatially patterned collagen scaffold, in conjunction with rehabilitative exercise, for treatment of volumetric muscle loss. To mimic the physiologic organization of skeletal muscle, which is generally composed of myofibers aligned in parallel, three-dimensional parallel-aligned nanofibrillar collagen scaffolds were fabricated. When implanted into the ablated murine tibialis anterior muscle, the aligned nanofibrillar scaffolds, in conjunction with voluntary caged wheel exercise, significantly improved the density of perfused microvessels, in comparison to treatments of the randomly oriented nanofibrillar scaffold, decellularized scaffold, or in the untreated control group. The abundance of neuromuscular junctions was 19-fold higher when treated with aligned nanofibrillar scaffolds in conjunction with exercise, in comparison to treatment of aligned scaffold without exercise. Although, the density of de novo myofibers was not significantly improved by aligned scaffolds, regardless of exercise activity, the cross-sectional area of regenerating myofibers was increased by > 60% when treated with either aligned and randomly oriented scaffolds, in comparison to treatment of decellularized scaffold or untreated controls. These findings demonstrate that voluntary exercise improved the regenerative effect of aligned scaffolds by augmenting neurovascularization, and have important implications in the design of engineered biomimetic scaffolds for treatment of traumatic muscle injury.

## Muscle injury: exercise boosts regenerative potential of engineered collagen scaffolds

A collagen scaffold designed to mimic skeletal muscle, together with rehabilitative exercise, can help regenerate nerves and blood vessels following traumatic muscle injury. Ngan Huang from Stanford University, California, USA, and colleagues created scaffolding composed of collagen nanofibers aligned in parallel, as natural muscle fibers are. They implanted these specially patterned collagen constructs into the shins of mice that had no tibialis anterior muscles. Mice given the opportunity to exercise formed far more nerve connections in their injured muscles compared to mice without exercise wheels in their cages. Active mice also developed significantly more blood vessels in their injured muscles with the parallel-aligned scaffolds compared to other animals with randomly oriented scaffolds, decellularized scaffolds or no implant at all. The findings highlight the potential of combining exercise and biomimetic scaffolds to treat muscle trauma.

## Introduction

Although skeletal muscle generally has high regenerative capacity, muscle regeneration can be permanently impaired by traumatic injuries. Volumetric muscle loss (VML) is characterized by the removal of a significant portion of skeletal muscle,^1^ leading to irreversible loss of muscle function as well as cosmetic deformity. There is no well-established standard of care for individuals with VML. Approaches to treat VML by autologous muscle flap transplantation or tissue debridement has shown limited benefit and donor site morbidity.^2–4^ It has become increasingly appreciated that recovery from VML also requires revascularization^5^ and innervation.^6^ Being highly metabolic, skeletal muscle is closely approximated to capillaries as a source of nutrients and oxygen to maintain muscle function and viability. Besides revascularization, innervation of the newly regenerated muscle is important for long-term functional integration and restoration of VML injury.^7^

Tissue engineering has emerged as a strategy to stimulate muscle regeneration. Biological scaffolds derived from naturally derived extracellular matrices (ECMs) can be implanted into the site of VML injury to provide structural support as well as promote cellular infiltration into the scaffold. Biological scaffolds derived from decellularized ECMs have been examined in both preclinical^8–11^ and clinical^12,13^ settings of VML. Recently, we showed that the addition of rehabilitative exercise can augment the function of engineered tissues in a preclinical model of VML,^14^ suggesting that administration of exercise may benefit implanted engineered tissues.

In physiological tissues, the ECM secreted by skeletal muscle and vascular cells form nano- to micro-scale fibrillar networks that align along the direction of the myofibers.^15–17^ We previously demonstrated that cues from aligned nanofibrillar ECMs not only modulate cell alignment, but also biological processes such as cell migration, angiogenesis, and cell survival.^18–22^ These findings are supported by a foundation of knowledge that spatially patterned ECMs regulate cellular morphology, tissue morphogenesis, and function.^22–25^

Recent studies suggest that rehabilitative exercise may be beneficial for treatment of VML. Voluntary caged wheel exercise was shown to improve force transmission in regenerated muscle after VML.^26^ We have previously demonstrated that a voluntary caged wheel exercise regimen after implantation of decellularized scaffolds seeded with muscle stem cells resulted in the formation of more mature neuromuscular junctions, greater force production, and increased revascularization, when compared to implantations of bioconstructs in the absence of exercise.^14^ In addition, decellularized scaffold implantation into injured human muscle with exercise intervention showed improved functional outcomes in strength and range of motion.^27^ Based on these studies, exercise appears to provide important mechanical signaling cues to promote constructive remodeling.

In this report, we employed three-dimensional (3D) parallel-aligned nanofibrillar scaffold aggregates and voluntary rehabilitative exercise to augment angiogenesis and muscle innervation in a mouse model of VML. Based on histological analysis, we show that the implantation of aligned nanofibrillar scaffolds, in conjunction with exercise, significantly improved vascular perfusion and innervation, compared to randomly oriented scaffolds or decellularized scaffolds.

## Results

### Mechanical characterization of aligned nanofibrillar scaffolds

**Fig. 1:**
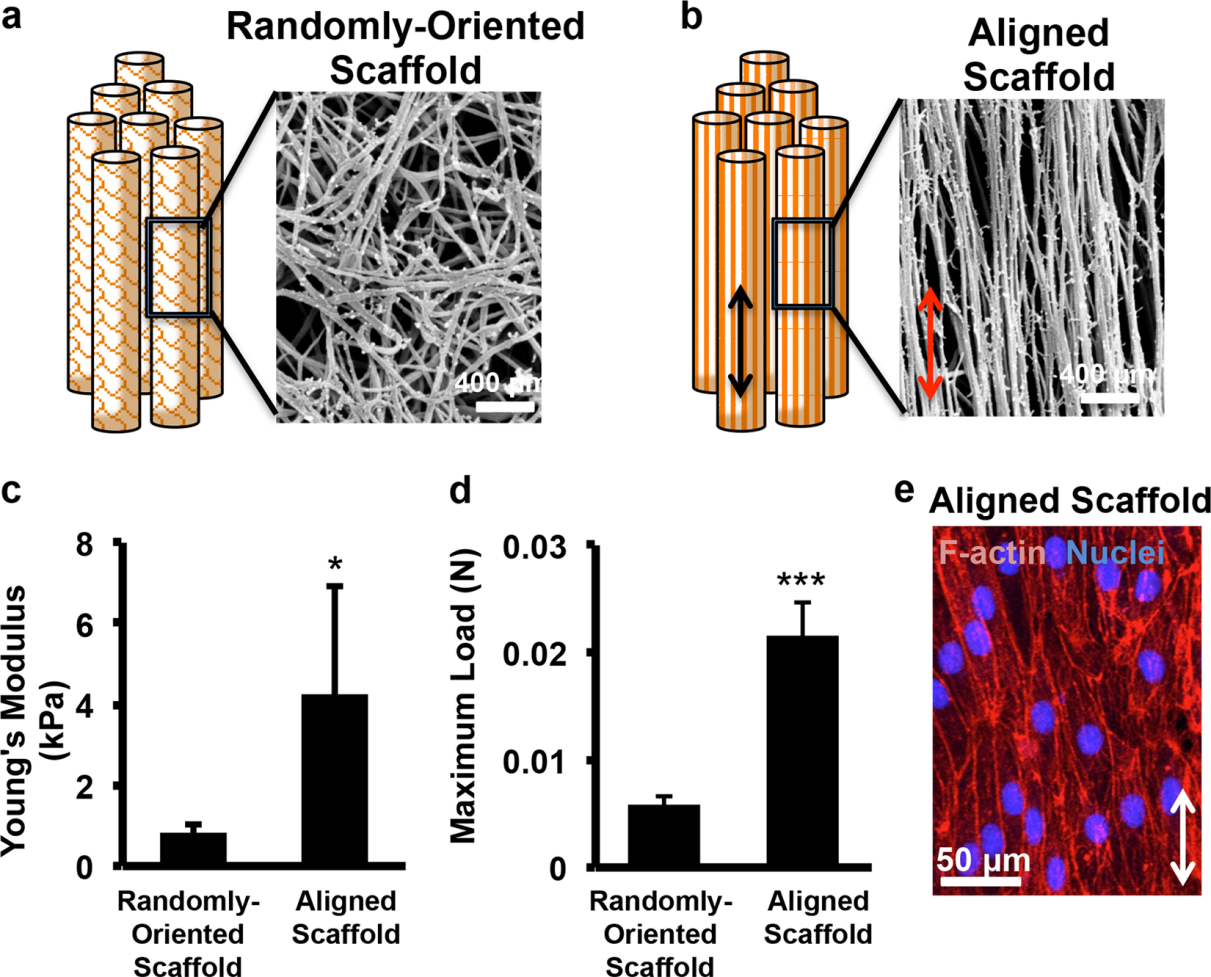
Characterization of aligned nanofibrillar collagen scaffold aggregates. **a** Schematic diagram of three-dimensional randomly oriented scaffold bundle and scanning electron microscopy (SEM) image of nanofibril organization. **b** Schematic diagram of three-dimensional parallel-aligned scaffold bundle and SEM image of nanofibril organization. **c, d** Mechanical characterization of stiffness by Young’s Modulus **c** and maximum load **d** between randomly oriented scaffolds (random) and aligned scaffolds (*n* = 5). **e** Cellular attachment of human endothelial cells to aligned nanofibrillar scaffolds. Scale bars: 400 µm **a**, **b**, 50 µm **e**. Arrow denotes the direction of nanofibril alignment. Statistically significant comparisons: * *P* < 0.05, ***P < 0.001. Error bars denote standard deviation

Individual strips of aligned or randomly oriented nanofibrillar scaffolds composed of collagen were fabricated by a shear-based extrusion approach (Fig. 1a–b), as described previously.^21,28,29^ To better mimic the physiological orientation of native muscle bundles, eight scaffolds were aggregated together, forming a 3D scaffold with dimensions of 9 mm × 2 mm × 3 mm. Scanning electron microscopy (SEM) imaging confirmed the parallel-aligned orientation of nanofibrils within each aligned nanofibrillar scaffold, with nanofibril diameters of 50 nm (Fig. 1b). In contrast, the randomly oriented scaffolds produced nanofibrils that were disorganized (Fig. 1a). Mechanical characterization of the 3D scaffold aggregates demonstrated that the aligned scaffolds were significantly stiffer, based on a Young’s Modulus of 4.3 ± 2.7 kPa, compared to that of randomly oriented scaffolds (0.9 ± 0.2 kPa, Fig. 1c, *P* < 0.05). Furthermore, aligned scaffolds could withstand more than three times greater load than 3D randomly oriented scaffold aggregates without failing (Fig. 1d, *P* < 0.001), suggesting that the aligned scaffold aggregate had higher tensile strength. The stiffness of scaffolds was within the range of Young’s Modulus reported for skeletal muscle (~12 kPa).^30^ To confirm the biocompatibility of the collagen scaffolds in vitro, primary human microvascular endothelial cells were seeded into the aligned scaffolds, and was shown to support robust cellular attachment, based on F-actin staining (Fig. 1e). Furthermore, when seeded with murine myoblasts in the presence of media promoting fusion, the cells formed multi-nucleated myotubes on aligned scaffolds that were generally organized along the direction of the nanofibril orientation (Supp Fig. 1).

### Aligned scaffolds for treatment of VML

After characterizing the mechanical properties and biocompatibility of the scaffolds, we implanted a 3D aggregate composed of eight scaffold strips in parallel into a murine model of acute VML. After excision of 20% of the tibialis anterior muscle, the scaffolds were transplanted into the void space of the ablated muscle. Animals were randomized to receive either aligned or randomly oriented scaffolds. After 3 weeks of implantation, the animals were systemically injected with isolectin that preferentially binds to perfused vessels, and the tibialis anterior muscles were then excised for immunofluorescence staining of skeletal muscle myosin heavy chain (MHC) for myofibers. As shown in Suppl. Figure 2, the aligned and randomly oriented scaffolds showed no significant difference in vascular perfusion (Suppl. Figure 2A), or muscle regeneration (Suppl. Figure 2B-C). This finding suggested that other stimulatory factors may be necessary for promoting muscular regeneration.

### Effect of voluntary exercise and implanted aligned scaffolds on revascularization

Since exercise has been shown by us and others to improve muscle function following VML,^14,26,27^ we hypothesized that voluntary exercise could augment the regenerative quality of implanted aligned nanofibrillar scaffolds by boosting de novo myogenesis, revascularization, and innervation. Based on our previous finding that mice with VML had restored daily running habits at 7 days after induced VML,^14^ we introduced a regimen in which the mice were randomized for voluntary caged wheel exercise from days 7 to 21 after implantation of either randomly oriented or aligned scaffolds. On day 21, the scaffold-implanted tibialis anterior muscle was quantitatively assessed for revascularization, muscle regeneration, and innervation. Animals without rehabilitation remained in traditional cages without caged wheels for the entire duration of the study. Animals showed no differences in averaged running distance (meters per day), regardless of treatment group (Suppl Fig. 3).

**Fig. 2:**
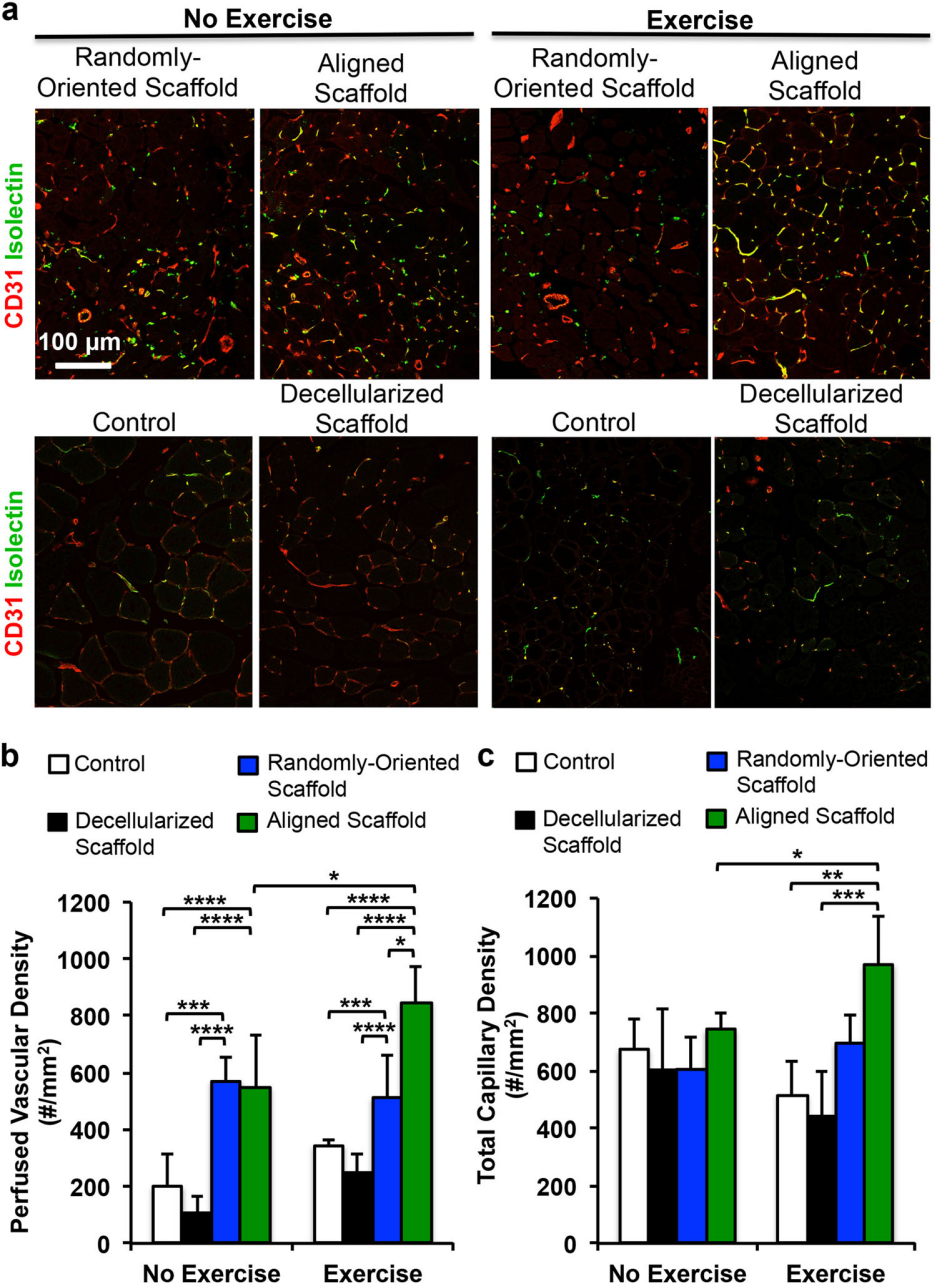
Therapeutic effect of aligned nanofibrillar scaffold with exercise on revascularization in the ablated muscle. **a** Confocal microscopy images adjacent to the site of scaffold implantation depict CD31 (red) and isolectin (green). **b** Perfused vascular density was quantified based on the density of CD31^+^/isolectin^+^ vessels. **c** Total capillary density was quantified as the density CD31^+^ vessels. Shown are mean ± SD aligned scaffold without exercise (*n* = 6), randomly oriented scaffold with exercise (*n* = 6), and all other groups (*n* = 4). Statistically significant comparisons: *P < 0.05, **P < 0.01, ***P < 0.001, ****P < 0.0001. **a** Scale bar: 100 µm. Error bars denote standard deviation

Tissue cross sections were stained by hematoxylin and eosin (H&E) or trichrome stains to visualize the morphology of the scaffold and the surrounding tissue. As shown in Suppl Fig. 4, remnants of the scaffolds could be visualized in all treatment groups. Immunofluorescence analysis of revascularization was performed by quantification of perfused vessels and total vessels. Perfused vessels were denoted as those that were fluorescently labeled by both isolectin and the endothelial marker, CD31 (Fig. 2a). Quantification of perfused vascular density within a 500 µm distance from the periphery of the scaffold demonstrated that the mice treated with aligned scaffolds and voluntary exercise had more than 50% greater perfused vascular density (850 ± 130/mm^2^) than mice treated with aligned scaffold without exercise (550 ± 180/mm,^2^ *P* < 0.05) or randomly oriented scaffolds with exercise (510 ± 150/mm,^2^ *P* < 0.05, Fig. 2b). Total vascularity density, comprising perfused as well as non-perfused vessels, similarly showed that treatment of aligned scaffold with exercise was significantly higher than treatment of aligned scaffolds without exercise (Fig. 2c, *P* < 0.05). Furthermore, when compared to animals receiving no treatment or decellularized scaffold treatment followed by voluntary exercise, animals with treatment of aligned scaffolds and exercise had > 80% higher perfused vascular density as well as total vascular density (*P* < 0.01). These findings suggested that voluntary exercise could boost revascularization in aligned scaffolds. Moreover, in conjunction with exercise, treatment of aligned scaffolds resulted in significantly improved revascularization, compared to treatment of randomly oriented scaffolds or decellularized scaffolds.

### Voluntary exercise and implanted aligned scaffolds on de novo myogenesis and innervation

**Fig. 3:**
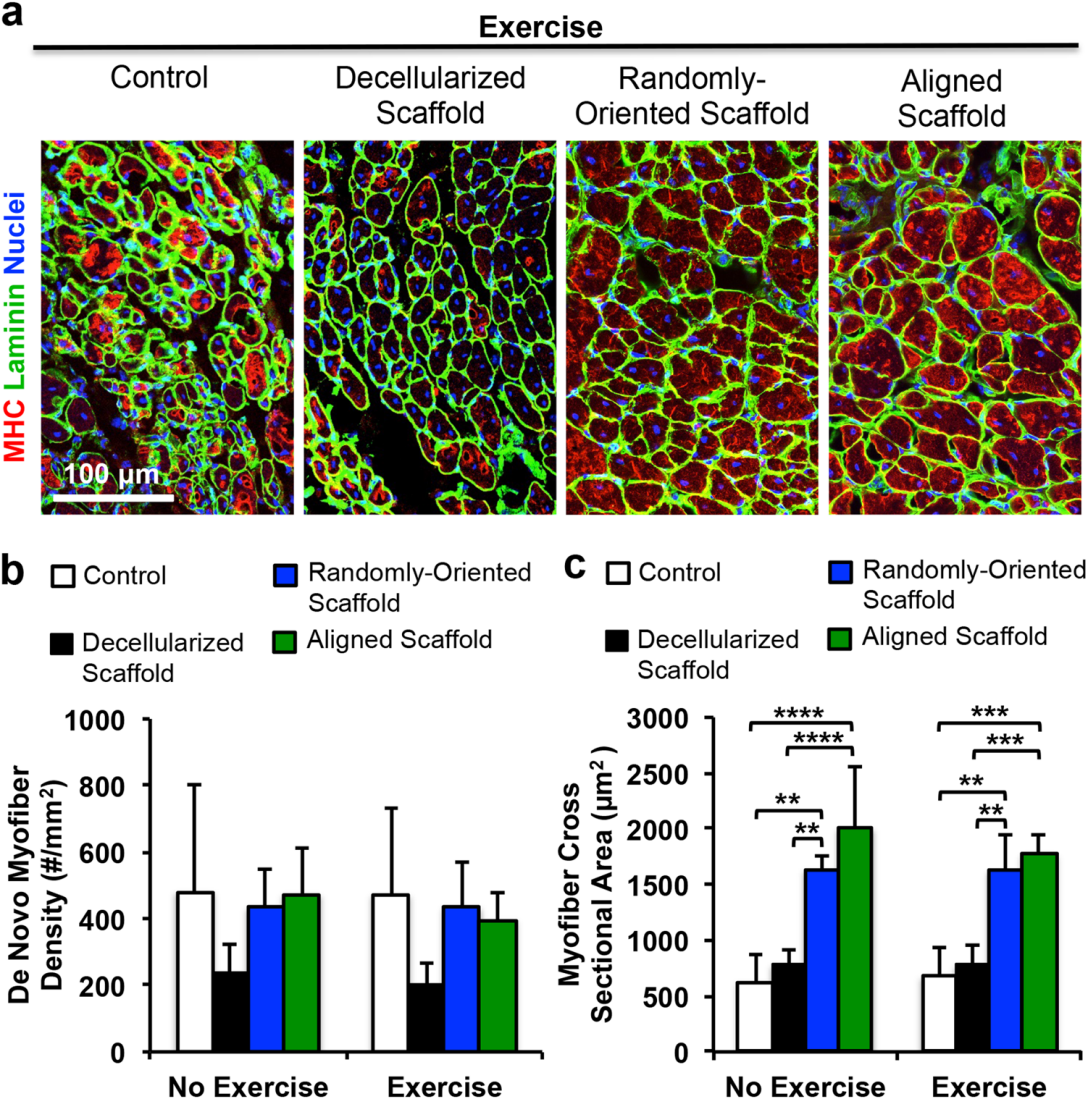
Effect of aligned nanofibrillar scaffold and exercise on myogenesis. **a** Confocal microscopy images adjacent to the site of scaffold implantation depicting regenerated myofibers based on antibodies against skeletal muscle myosin heavy chain (MHC, red) and laminin (green). **b** Quantification of myogenesis based the density of MHC^+^ myofibers with centrally located nuclei. **c** Quantification of myofiber cross-sectional area among regenerated myofibers. Shown are mean ± SD (aligned scaffold with or without exercise (*n* = 6), randomly oriented scaffold with exercise (*n* = 5), and all other groups (*n* = 4)). Statistically significant comparisons: *P < 0.05, **P < 0.01, ***P < 0.001, ****P < 0.0001. **a** Scale bar: 100 µm. Error bars denote standard deviation

We next assessed the potential benefits of aligned nanofibrillar scaffolds and voluntary exercise on myogenesis. De novo myofibers could be visualized by confocal microscopy based on the expression of MHC and centrally located nuclei. Within a 500 µm distance around the scaffold, the density of regenerated myofibers was quantified for each treatment group. As shown in Fig. 3a–b, there was no significant difference in myogenesis between aligned and randomly oriented scaffolds, regardless of exercise activity. Immunofluorescence staining of laminin was used to delineate the boundaries between myofibers for quantification of myofiber cross-sectional area. Cross-sectional area analysis of regenerated myofibers revealed an average of 1770 ± 140 µm^2^ in mice after treatment of the aligned scaffold with exercise, which was not significantly different from animals treated with randomly oriented scaffolds with exercise (Fig. 3c). However, the cross-sectional area of de novo myofibers after treatment of aligned or randomly oriented scaffolds was 60% greater compared to non-treated controls (670 ± 560 µm^2^) and decellularized scaffold controls (780 ± 170 µm^2^) with exercise (*P* < 0.01). As shown in Suppl Fig. 5, the histogram of cross-sectional area distribution shows significantly higher frequency of small (<500 µm^2^) myofibers in mice without treatment or mice receiving decellularized scaffold treatment, compared to treatment of nanofibrillar scaffolds (*P* < 0.05). In contrast, there was a significantly higher frequency of larger (>500 µm^2^) myofibers in animals treated with aligned or randomly oriented scaffolds, compared to animals with treatment of decellularized scaffold or untreated animals (*P* < 0.05). However, no statically significant differences were detected in myofiber cross-sectional areas, when comparing any treatment or control group without exercise, compared to the same group in which exercise was introduced. The degree of fibrosis was not significantly different between treatment of aligned or randomly oriented scaffolds, regardless of exercise activity (Suppl Fig. 6).

**Fig. 4:**
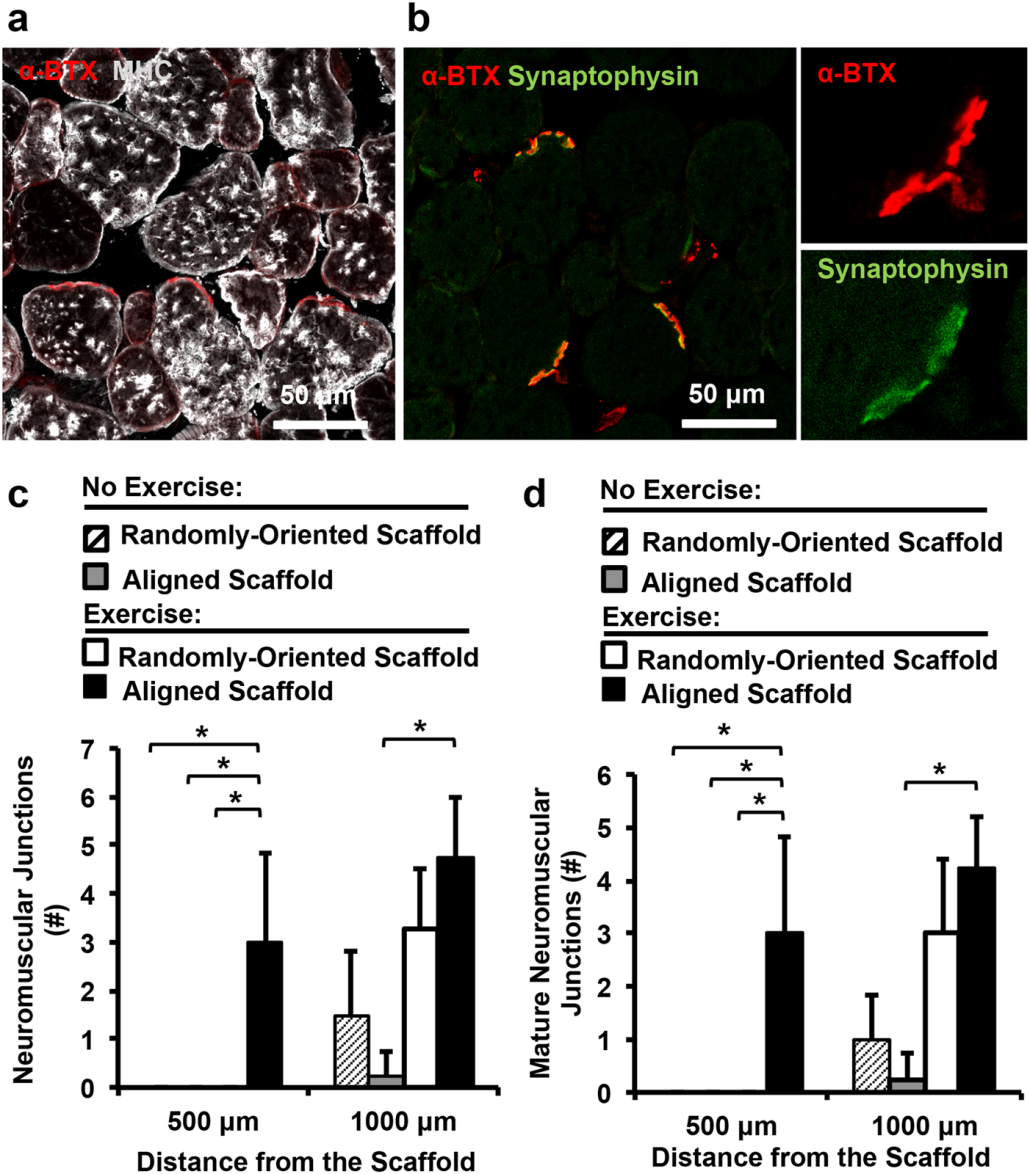
Effect of aligned nanofibrillar scaffold and exercise on re-innervation. **a**, **b** Confocal microscopy images adjacent to the site of scaffold implantation depicting re-innervation, based on α-bungarotoxin (α-BTX) and synaptophysin (green). **c** Quantification of re-innervation by the total number of neuromuscular junctions (α-bung^+^) at 500 µm or 1000 µm distance surrounding the scaffold. **d** Quantification of mature neuromuscular junctions (α-bung^+^/ synaptophysin^+^) at 500 µm or 1000 µm distance surrounding the scaffold. Shown are mean ± SD (*n* = 4 all groups). * Statistically significant comparisons (*P < 0.05). **a, b** Scale bar: 50 µm. Error bars denote standard deviation

Although the degree of myogenesis was not improved, we hypothesized that exercise might have a benefit on re-innervation of the muscle fibers, based on our previous study.^14^ The neuromuscular junctions within the vicinity of the scaffold were quantitatively assessed by fluorescence imaging of α-bungarotoxin (αBTX) (Fig. 4a, c). Confocal microscopy imaging revealed that neuromuscular junctions within a 500 µm distance from the transplanted scaffold were formed only when the animals were treated with aligned scaffold in conjunction with exercise (Fig. 4c). When expanding to a 1000 µm distance from the transplanted scaffold, the treatment group consisting of the aligned scaffold with exercise produced a 19-fold increase in neuromuscular junctions, compared to treatment of the aligned scaffold without exercise (*P* < 0.05). Furthermore, the number of mature neuromuscular junctions was quantitatively assessed by dual staining of αBTX and synaptophysin (Fig. 4d). The results similarly revealed that exercise could significantly improve the abundance of mature (αBTX^+^/synaptophysin^+^) neuromuscular junctions at 500 µm and 1000 µm distances from the scaffold. Together these results suggest that exercise could boost the therapeutic benefit of aligned scaffolds by increasing revascularization and re-innervation.

## Discussion

The salient finding of this study was that treatment of aligned scaffolds in conjunction with rehabilitative voluntary exercise improved revascularization, compared to treatment of randomly oriented scaffolds, decellularized scaffolds, or no treatment (Fig. 2). Furthermore, treatment of aligned scaffolds in conjunction with exercise led to significantly more mature neuromuscular junctions, compared to the absence of exercise (Fig. 4). Although de novo myofiber density was not significantly improved by treatment of aligned nanofibrillar scaffolds, myofiber cross-sectional area was significantly higher in animals treated with aligned scaffolds, compared to no treatment or treatment with decellularized scaffolds, regardless of exercise activity (Fig. 3).

The aligned nanofibrillar collagen scaffolds recapitulate the ordered arrangement of fibrillar collagen in skeletal muscle.^16^ Patterned biomaterials have been shown to improve tissue regeneration and reduced scar tissue deposition.^31^ Aligned scaffolds could potentially act in concert with exercise to promote angiogenesis, expedite regeneration, or confer spatial guidance cues to surrounding cells longitudinally along the length of the muscle, thereby improving organization and efficiency of revascularization and re-innervation. Based on the higher degree of tensile strength in the aligned nanofibrillar scaffolds, compared to the randomly oriented scaffolds, it is plausible that these differences in mechanical characteristics could influence the amount of effective force transmission to the muscle during the rehabilitative exercise period. Furthermore, the Young’s Modulus of the aligned scaffold resembles the substrate stiffness (~2 kPa) that we previously showed to be optimally conducive to muscle stem cell potency.^32^ Although the mechanosignaling mechanisms are largely unknown, exercise might provide constructive signaling cues to aligned scaffolds to direct neurovascular regeneration. We previously demonstrated that aligned nanofibrillar scaffolds augmented greater endothelial outgrowth, in part, by preferential activation of integrin α1 signaling pathway,^20^ but it is unknown whether exercise-mediated mechanical stretching of the scaffolds can modulate integrin activation. Further studies are needed to evaluate long-term benefits and whether exercise preferentially activates mechanosignaling pathways in aligned nanofibrillar scaffolds during muscle regeneration to inform the necessary characteristics of therapeutic scaffolds.

As an off-the-shelf biomaterial with less demanding storage requirements than therapeutic cells, acellular ECM scaffolds may be an attractive option for treatment of VML. Currently, there are already a number of FDA-approved scaffolds indicated for soft tissue applications that was shown to be clinically effective for treatment of VML.^13^ However, many of these are xenogenic decellularized scaffolds or those derived from tissues other than skeletal muscle. Decellularized scaffolds can vary in their chemical composition, mechanical properties, and degradation rate. Furthermore, it is unclear whether decellularized scaffolds retain the nano- to micro-scale spatial organization of fibrillar ECMs that recapitulates that of physiological muscle. Due to the potential differences in mechanical properties, chemical composition, biodegradability, and method of decellularization, the potential therapeutic impact of decellularized scaffolds can vary.^33^ Despite some reports that decellularized scaffolds lead to constructive muscle regeneration,^12,13^ the decellularized porcine urinary bladder scaffold used in this study showed no significant improvement in vascular perfusion, de novo myogenesis and myofiber cross-sectional area, compared to the untreated control group. This finding agrees with published reports, in which implantation of decellularized scaffolds resulted in no improvement in vascular regeneration, innervation,^34^ and myogenesis,^35^ compared to the untreated control group.

A potential limitation of the study is that the kind of decellularized scaffolds used were xenogenic and not derived from skeletal muscle. Since decellularized scaffolds are predominantly composed of collagen I,^36^ the randomly oriented collagen scaffolds mimic many of salient features of decellularized collagen. However, differences between decellularized and engineered collagen scaffolds do exist with regard to mechanical and biophysical properties, including the loss of fibrillar ECM structure during the decellularization process.^36^ Evidence from this study suggests that randomly oriented scaffolds differ from decellularized scaffolds in having significantly greater degree of perfused vasculature and myofiber cross-sectional area, compared to decellularized scaffolds.

Towards improved de novo muscle regeneration, future work is aimed at addition of reparative and immunomodulatory cell populations to the aligned scaffolds, to address the limitations of acellular scaffold-mediated regeneration.^12,37^ Furthermore, future studies are required to evaluate the functional recovery of muscle and neuromuscular synapses in response to VML, as well as the ability of the scaffolds to sustain muscle regeneration when re-injured again. Validation of these findings in large animal models will also be an important future step towards clinical translation. Acellular scaffolds with suitable mechanical and biophysical properties have the potential to create a pro-regenerative environment that enhances muscle stem cell function and inflammatory response.^38^ Our previous study shows that exercise can enhance myogenesis in the setting of VML when muscle stem cells are transplanted to the site of muscle ablation,^14^ suggesting that transplanted muscle stem cells may have a greater therapeutic effect than endogenous cells. Together, therapeutic cells can act in concert with aligned nanofibrillar scaffolds to synergistically improve muscular and neurovascular regeneration associated with VML.

In summary, the results of this study show that aligned nanofibrillar scaffolds, in conjunction with voluntary caged wheel exercise, can facilitate functional tissue remodeling following VML by inducing revascularization and re-innervation, and by increasing de novo myofiber size. These findings have important implications in the design of engineered biomimetic scaffolds and exercise regimens for treatment of traumatic muscle injury.

## Methods

### Generation of parallel-aligned nanofibrillar collagen scaffolds

Fabrication of aligned nanofibrillar collagen scaffold strips was described previously.^29^ Briefly, rat-tail collagen-Type I (10 mg/mL in 0.02 N in acetic acid, pH 3.5, Corning) was dialyzed to 30 mg/mL using a semi-permeable cellulose dialysis tubing of pore size 32 × 20.4 mm (Thermo Fisher) and polyethylene glycol (Sigma). The aligned nanofibrillar collagen scaffold strip (25 mm × 1 mm) was extruded from a 22 G blunt tip needle onto glass slides at high velocity (340 mm/s), submerged within 10X phosphate-buffered saline (PBS, pH 7.4) at 37 °C to initiate fibrillogenesis instantaneously as the collagen is extruded. Randomly oriented scaffold strips of a similar size were made by extrusion at a decreased speed. To create a 3D scaffold bundle, eight scaffold strips were aggregated in parallel with dimensions that were 9 mm × 2 mm × 3 mm (Fig. 1a–b). The nanostructure and fibril alignment of the scaffolds were visualized by routine SEM, as described previously.^29^

### Mechanical characterization

The 3D scaffold aggregates of randomly oriented of aligned scaffolds were rehydrated with deionized water for 30 min, prior to testing. The scaffolds (*n* = 5) were clamped at their cut ends in Universal Testing Machine (Instron 5565). The tensile force applied to constantly stretch the scaffold at 0.2 mm/second was measured and recorded using a 100 N maximum load cell, and the test was stopped when the load decreased after the onset of failure. The maximum load (N) was identified from the load-displacement curve. From the stress–strain graph produced, the Young’s modulus (kPa) was calculated as the slope of the elastic region of the curve.

### Cellular attachment onto scaffolds

To assess for cytotoxicity of the scaffolds, randomly oriented or aligned collagen scaffolds were disinfected with 70% ethanol and rehydrated with phosphate-buffered saline (PBS) for 2 h. Primary human dermal microvascular endothelial cells (0.5 × 10^6^ cells, Lonza) were seeded per collagen scaffold and cultured in EGM-2 MV growth media (Lonza) at 37 °C and 5% CO2 until they reached approximately 80% confluence. After 24 h, the cell-seeded scaffolds (*n* = 3 each) were fixed in 4% paraformaldehyde and samples were stained for F-actin using Alexa Fluor-488-conjugated phalloidin (Life Technologies). Samples were then counterstained with Hoechst33342 to visualize nuclei and imaged in the hydrated state in PBS. Scaffolds were imaged using confocal microscopy (Zeiss LSM 770). To examine the ability of aligned nanofibrillar scaffolds to modulate cellular organization, green fluorescent protein-tagged mouse myoblasts (C2C12, ATCC) were seeded onto the scaffolds and allowed to fuse for 5 days in Dulbecco’s Modified Eagle’s Medium (DMEM) containing 2% horse serum. Cells on the scaffolds were imaged live using confocal microscopy (Zeiss LSM 770).

### Transplantation of 3D nanofibrillar scaffold aggregate into a mouse model of VML

All animal studies were approved by the Institutional Animal Care and Use Committee at the Veterans Affairs Palo Alto Health Care System. VML was induced in immunocompromised NOD-*scid* IL2Rg^null^ mice (male, 8 weeks old, Jackson Laboratory) by surgical excision of 20% of the tibialis anterior (TA) muscle bilaterally, excising a segment that was 7 mm × 2 mm × 3 mm.^14,39^ Immediately afterwards, animal were randomized to receive one of the following treatment groups per legat the site of the muscle defect: (1) no treatment (*n* = 4); (2) randomly oriented scaffold aggregate (*n* = 6); (3) aligned scaffold aggregate (*n* = 6); or (4) commercial decellularized scaffolds (Gentrix, Acell Corporation, *n* = 4). Each scaffold aggregate was composed of eight scaffold strips organized as a parallel bundle with dimensions of 9 mm × 2 mm × 3 mm. The decellularized scaffold served as a basis for comparison to nanofibrillar scaffolds. Following scaffold transplantation, animals were allowed to recover in traditional housing cages for 21 days before being euthanized for histological analysis.

### Rehabilitative exercise regimen

In separate studies, following scaffold transplantation, animals were allowed to recover in traditional housing cages for 7 days. After 7 days of rest, animals from each group were moved to individual cages containing cage wheels (Lafayette Inst) and randomized into the following groups: aligned scaffold (*n* = 6), randomly oriented scaffold (*n* = 6), decellularized scaffold (*n* = 4), or no treatment (*n* = 4). Each cage wheel was attached to an electronic counter that interfaced with a computer that recorded the time and distance traveled every 15 s via Scurry 17.9 software (Layfayette Instruments). Animals were exercised for 14 days (days 7 to 21 days post transplantation). All animal studies were approved by the Institutional Animal Care and Use Committee at the Veterans Affairs Palo Alto Health Care System.

### Histological analysis of blood perfusion

On day 21 after scaffold implantation, animals were injected via the tail vein with 100 µL (1 mg/mL) of endothelial-binding fluorescent isolectin GS-IB4 (Invitrogen) prior to euthanasia. The TA muscle was then explanted and fixed in 0.4% paraformaldehyde at 4 °C for 16 h, followed by density equilibration in 20% sucrose for 2 h and embedding for cryosectioning of tissue sections in the transverse or longitudinal planes. Histological quantification of blood perfusion was performed by immunofluorescence staining of endothelial marker, CD31 (R&D Systems). Five non-overlapping images (500 µm x 500 µm) from transverse cryosections for each animal were taken within a 500 µm radius from the transplanted scaffolds (aligned scaffold without exercise (*n* = 6), randomly oriented scaffold with exercise (*n* = 6), and all other groups (*n* = 4)). The CD31^+^ vessels that co-stain with isolectin indicated vessels with functional anastamosis to the host circulation. The perfused vessel density was expressed as the total number of perfused vessels per square millimeter.

### Immunofluorescent staining and assessment of muscle regeneration

Serial transverse cryosections (10 µm thickness) of the TA muscle were stained with routine H&E or Trichrome stains to examine tissue morphology. To quantify myofiber regeneration around the scaffold, muscle tissue cryosections were immunofluorescently stained with MHC and tiled z-stacked images (5 × 5 montages using 20X objectives) were taken using confocal microscopy (Zeiss LSM880). Using the multipoint tool in ImageJ, the total number of MHC^+^ myofibers with centrally located nuclei in the vicinity of the scaffold implants (defined as within 500 µm from the scaffold periphery) was counted (aligned scaffold with or without exercise (*n* = 6), randomly oriented scaffold with exercise (*n* = 5), and all other groups (*n* = 4)). In this same region, using the area and circumference tool in ImageJ, the myofiber area was assessed. Fibrosis was quantified by collagen content based on Trichrome staining, and expressed as the % of area within 500 µm from the scaffold periphery (*n* = 4).

### Immunofluorescent staining and assessment of muscle innervation

Histological quantification of muscle innervation was performed by immunofluorescence staining of neuromuscular junction markers, α-bungarotoxin (Invitrogen) and synaptophysin (Sigma). Five non-overlapping images (500 µm x 500 µm) from transverse cryosections for each animal (*n* = 4 each group) were taken within 500 µm or 1000 µm radius from the transplanted scaffolds. The number of α-bungarotoxin^+^ and synaptophysin^+^ neuromuscular junctions were quantified to give the mature neuromuscular junction density (# α-bungarotoxin^+^/synaptophysin^+^ junctions per square millimeter). The density of total neuromuscular junctions was defined as the # α-bungarotoxin^+^ junctions per square millimeter).

### Statistical analysis

All statistical analysis was performed using Graph Pad PRISM software. For comparison between two groups, an unpaired *t*-test was performed. Where appropriate, a one-way ANOVA or two-way ANOVA was performed with post hoc Tukey’s adjustment. Significance was taken at *P* ≤ 0.05 (*), *P* ≤ 0.01 (**), *P* ≤ 0.001 (***), and *P* ≤ 0.0001 (****). All graphs were made in either Microsoft Excel or GraphPad PRISM and display mean ± standard deviation.

## Disclaimer

The content is solely the responsibility of the authors and does not necessarily represent the official views of the National Institutes of Health, Department of Defense, or the Department of Veteran Affairs.

## Electronic supplementary material

Suppplemental Figures

## Data Availability

The datasets generated during and/or analysed during the current study are available from the corresponding author on reasonable request.

## Electronic supplementary material

**Supplementary information** accompanies the paper on the *npj Regenerative Medicine* website (10.1038/s41536-018-0054-3).
