# Supplementary material for: Rehabilitative exercise and spatially patterned nanofibrillar scaffolds enhance vascularization and innervation following volumetric muscle loss
Source: NPJ Regen Med. 2018 Sep 17;3:16. doi: 10.1038/s41536-018-0054-3 (PMC6141593; doi:10.1038/s41536-018-0054-3)
Supplement: Supplementary file 1 — Suppplemental Figures [file 41536_2018_54_MOESM1_ESM.pdf]

Supplementary Information

Rehabilitative Exercise and Spatially Patterned Nanofibrillar Scaffolds Enhance Vascularization and Innervation Following Volumetric Muscle Loss

Karina H. Nakayama[1,2,3], Cynthia Alcazar[1], Guang Yang,[1,2] Marco Quarta [1,4], Patrick Paine[1], Linda Doan[1], Adam Davies[1], Thomas A. Rando[1,4], Ngan F. Huang[1,2,3*]

Affiliations:
[1] Veterans Affairs Palo Alto Health Care System, 3801 Miranda Avenue, Palo Alto, CA, 94304
[2] The Stanford Cardiovascular Institute, Stanford University, Stanford, CA, 94305
[3] Department of Cardiothoracic Surgery, Stanford University, Stanford, CA, 94305
[4] Department of Neurology and Neurological Sciences, Stanford University, Stanford, CA, 94304

*To whom correspondence should be addressed:
Dr. Ngan F. Huang, PhD
Email: ngantina@stanford.edu

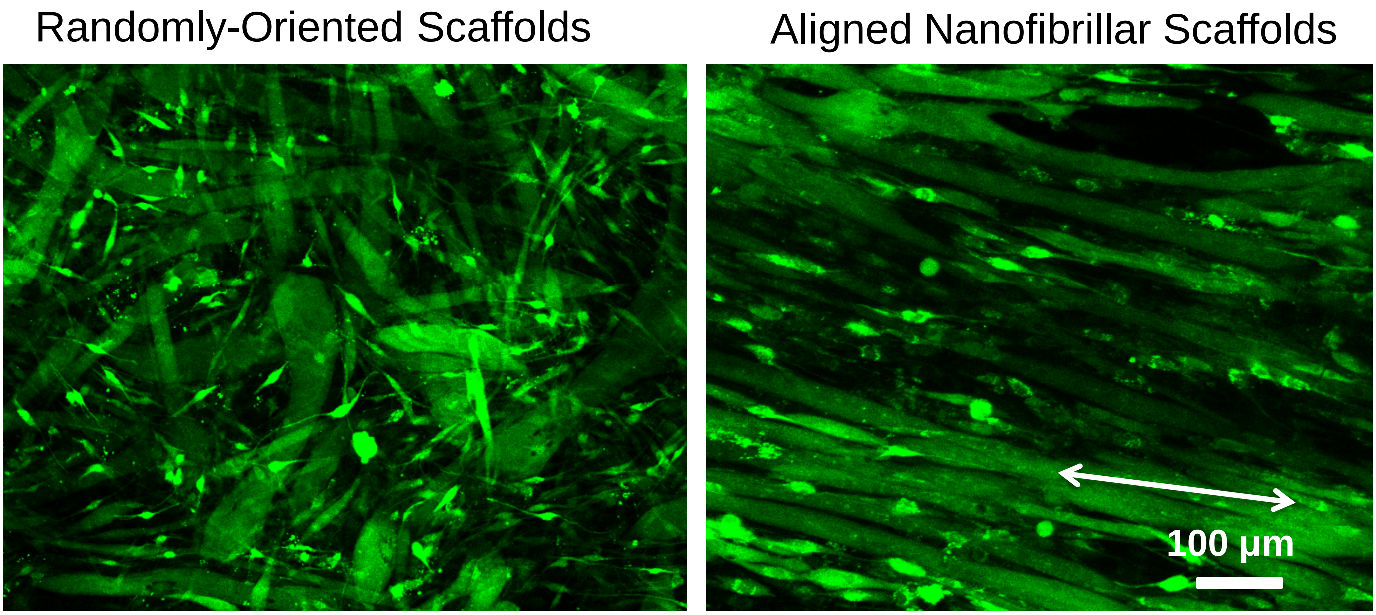

Supp Fig 1. Green fluorescence protein-expressing mouse myotubes formed on randomly oriented or aligned scaffolds after 5 days of differentiation. Double arrows depict orientation of aligned nanofibrils.

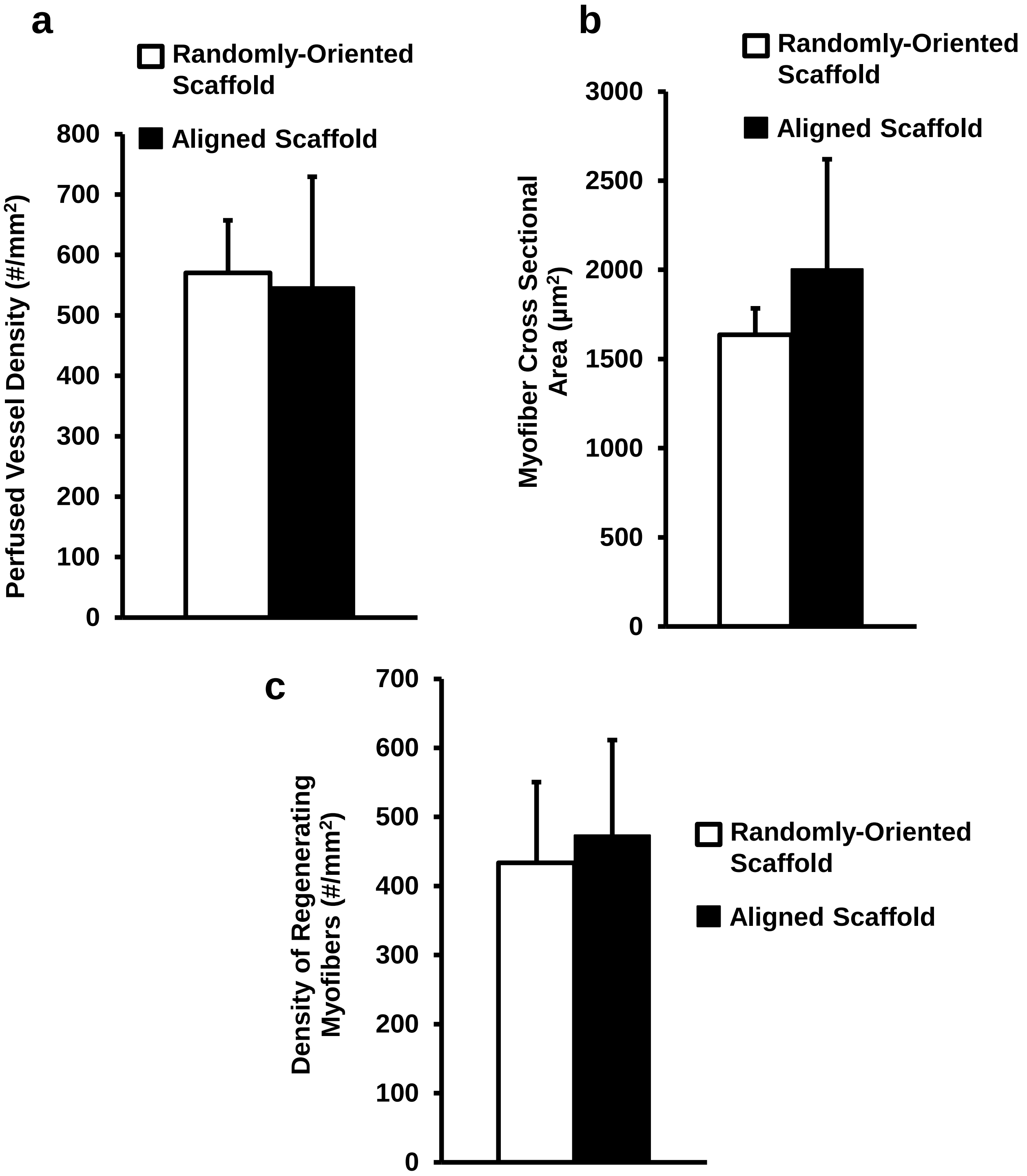

Supp. Fig. 2. Muscle regeneration and revascularization after implantation of aligned or randomly oriented scaffold aggregates in a mouse model of VML. A-C. Randomly oriented or aligned scaffolds were implanted into the void space of the ablated tibialis anterior muscle. After 3 weeks of implantation in the absence of exercise, immunofluorescence analysis was performed on the tibialis anterior muscle for antibodies against CD31, myosin heavy chain (MHC), and laminin. Within a 500µm distance from the edge of the scaffold, quantification of perfused ($CD31^+$/isolectin$^+$) vessel density (A), MHC^+ myofiber cross sectional area (B), and *de novo* myogenesis (# MHC^+ myofibers /mm^2) (C) were performed. Shown are mean ± SD (randomly oriented scaffold (n=4), aligned scaffold (n=6). Error bars denote standard deviation.

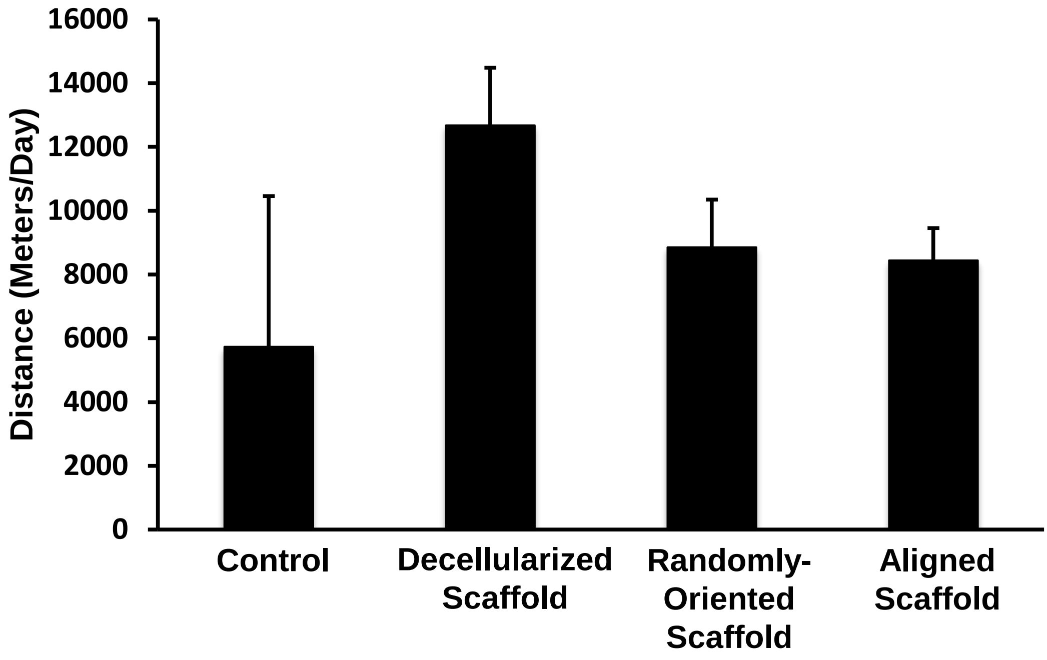

Suppl. Fig. 3. Caged Wheel running distance (meters). 7 days after initial transplants, mice were introduced to exercise wheels and running distance was tracked per day. Graph shows the averaged running distance per day for each treatment group (n=3 each group). Error bars denote standard deviation.

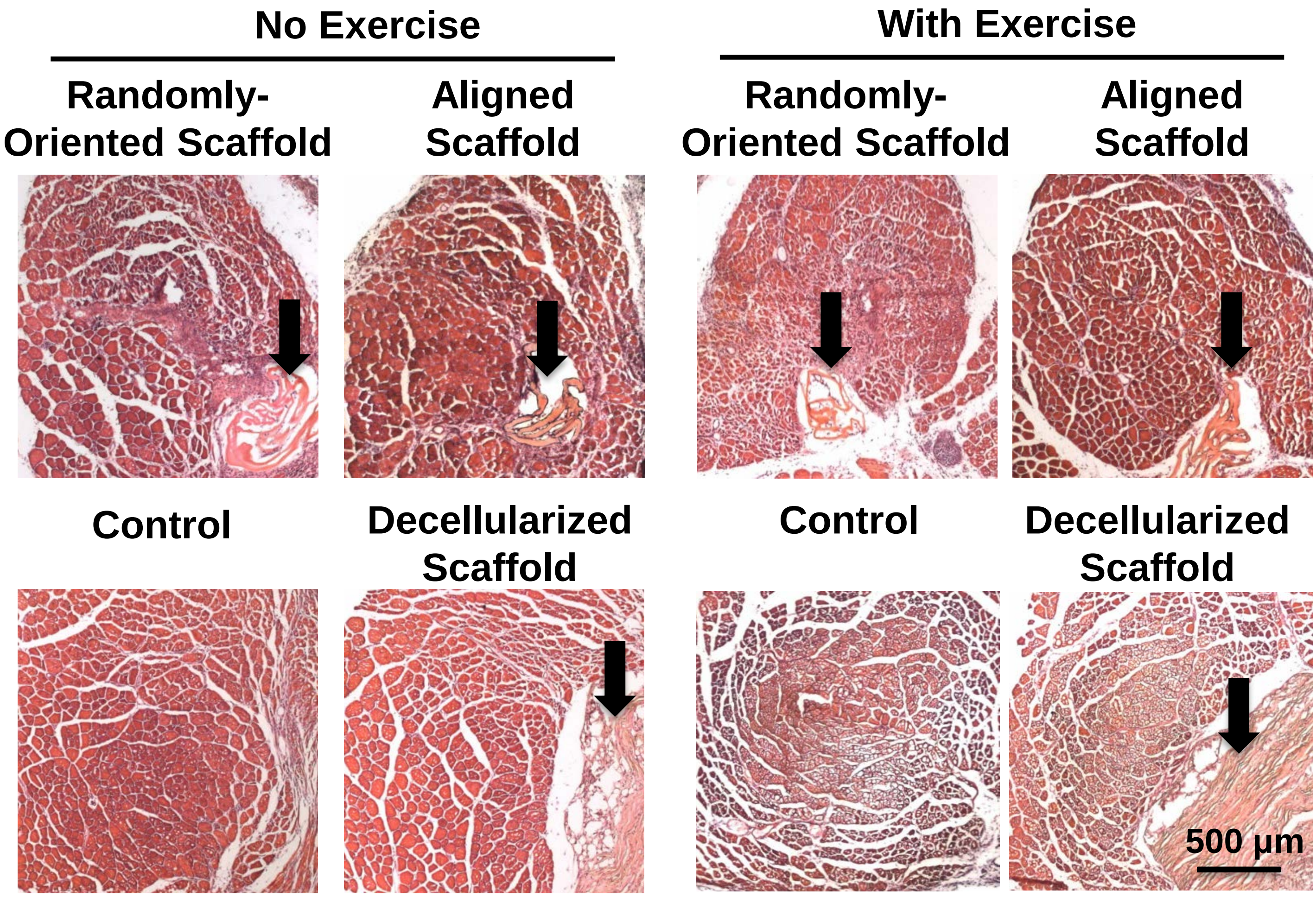

Suppl. Fig. 4. Histological assessment of tissue cross sections by hematoxylin and eosin stains. At 3 weeks after implantation into the ablated tibialis anterior muscle, the scaffold-containing muscle was excised. H&E staining was used to visualize the remnants of the scaffold (denoted by arrow). Scale bar: 500 μm.

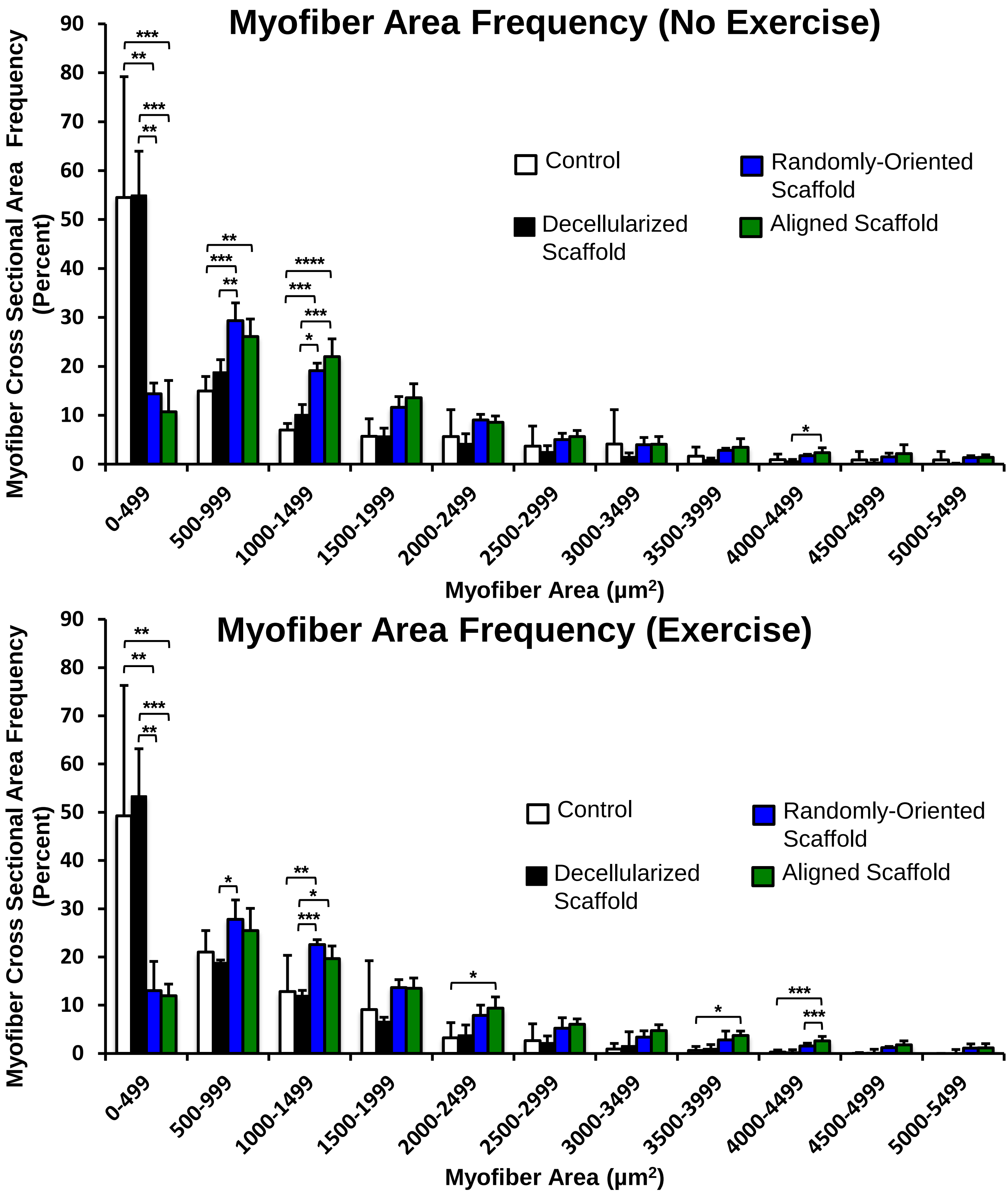

Suppl. Fig. 5. Histological assessment of regenerating myofiber cross-sectional area. Frequency distribution of mean myofiber area sizes in the presence or absence of exercise. Shown are mean ± SD (aligned scaffold with or without exercise (n=6), randomly oriented scaffold with exercise (n=5), and all other groups (n=4)). Statistically significant comparisons:*P<0.05, **P<0.01, ***P<0.001, ****P<0.0001. Error bars denote standard deviation.

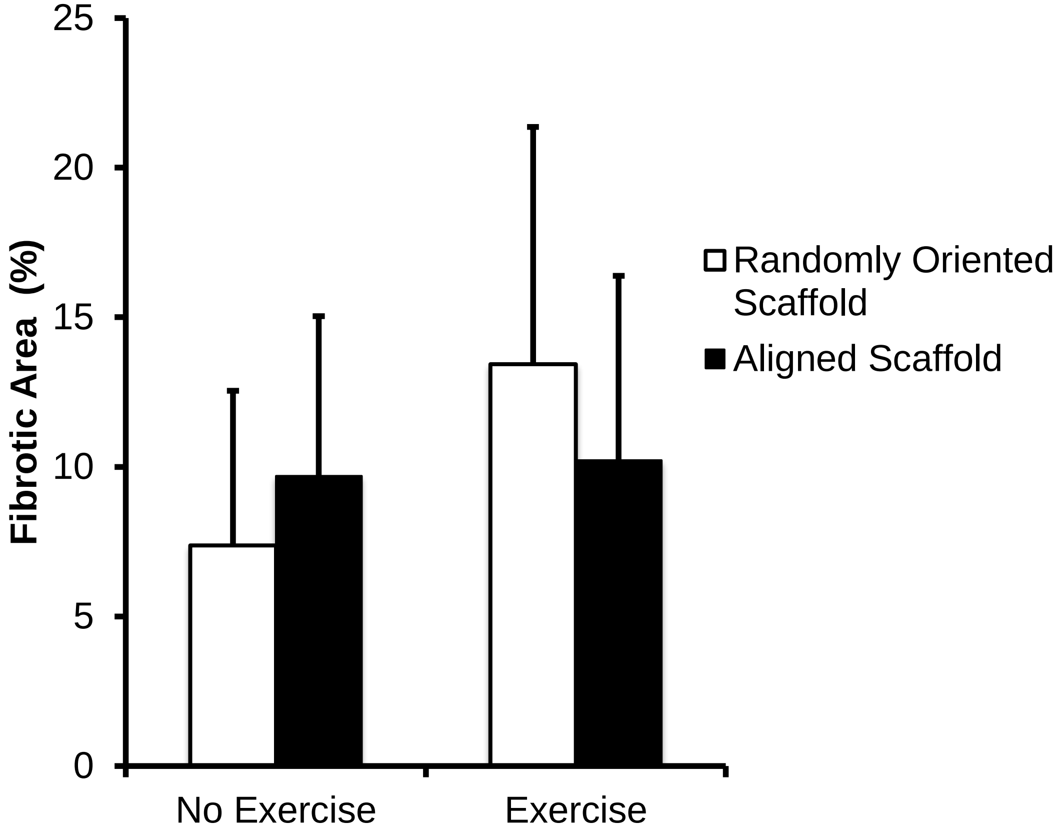

Suppl Fig. 6. Quantification of fibrosis in the injured muscle by the percent area of collagen from Trichrome-stained slides (n=4). Error bars denote standard deviation.
